# A Co‐Produced Stakeholder Workshop to Identify Key Time Points and Targets for Life‐Course Prevention of Multiple Long‐Term Conditions

**DOI:** 10.1111/hex.70475

**Published:** 2025-10-24

**Authors:** Sebastian Stannard, Rebecca Wilkinson, Jaskiran K. Gill, James McMahon, Jack Welch, Simon D. S. Fraser, Nisreen A. Alwan

**Affiliations:** ^1^ School of Primary Care, Population Sciences and Medical Education, Faculty of Medicine University of Southampton Southampton UK; ^2^ NIHR Applied Research Collaboration Southampton United Kingdom of Great Britain and Northern Ireland; ^3^ Southampton City Council Southampton United Kingdom of Great Britain and Northern Ireland; ^4^ Public Policy University of Southampton Southampton United Kingdom of Great Britain and Northern Ireland; ^5^ University of Southampton Southampton United Kingdom of Great Britain and Northern Ireland; ^6^ University Hospital Southampton NHS Foundation Trust Southampton United Kingdom of Great Britain and Northern Ireland

## Abstract

**Introduction:**

The MELD‐B project is a multidisciplinary research consortium with one of its aims focused on identifying childhood targets for the prevention of multiple long‐term conditions (MLTCs). Drawing upon the expertise of policy and practice stakeholders can inform research questions, data analysis, and contribute to meaningful and practical outputs. In pursuit of this collaborative approach, a stakeholder workshop, co‐designed with people with lived experience, was conducted to inform the next steps of the early prevention workstream of the MELD‐B project.

**Methods:**

The research team worked with four public contributors to co‐design the workshop in terms of its aims and structure. This involved utilising a project‐specific animation and developing an imaginary persona to illustrate the life‐course concepts of MLTCs, with an emphasis on how early life factors can influence outcomes later in life. Stakeholders were divided into three groups, each with a mix of professions and facilitated by two team members. Jamboard (an online interactive whiteboard) was used to collate ideas, and overarching themes were identified. A poll was administered at the end of the workshop giving choices to prioritise time points for interventions.

**Results:**

25 stakeholders with policy and practice expertise of childhood attended the workshop. Stakeholders were from backgrounds including integrated care boards (*n* = 5), healthcare practitioners (*n* = 3), academics (*n* = 4), council employees (*n* = 9) and not‐for‐profit organisations (*n* = 4). The workshop aimed to identify critical time points and targets in the early life‐course for feasible and practical interventions to prevent or delay MLTCs. Themes discussed included: mental health, educational attainment, early identification of health conditions and neurodiversity, nutritional choices, transitional periods, the virtual world, and intermediate outcomes on the pathway to future ill health. Stakeholders suggested that family‐targeted interventions were important to prioritise in early childhood; however, at secondary school age individual‐focused interventions may become more significant. A poll identified birth and ages 5–7 and 10–11 as the most important time points for interventions.

**Conclusions:**

People with lived experience should have central roles in shaping research questions, prioritising problems and engaging with stakeholders. Our workshop identified priority themes to inform prevention interventions using routinely collected and national cohort data.

**Patient or Public Contribution:**

The research team worked with four public contributors to co‐design the workshop in terms of its aims and structure. Public contributions helped to identify stakeholders to invite to the workshop and co‐produced PowerPoint slides to guide the workshop. In addition, the workshop utilised a co‐produced project‐specific animation and imaginary persona to help frame our research. Public contributors attended the workshop and helped to facilitate discussions by providing their own lived experience. Following the workshop, public contributors reviewed the themes identified by the research team from the discussions within the workshop. Finally, public contributors have also been involved in dissemination of the findings from this study, including this paper. Two of the public contributors are named co‐authors on this paper, and two did not wish to be named as co‐authors.

## 1 Introduction

Stakeholder engagement in research is defined as ‘an umbrella term for the types of research (e.g., patient‐centred outcomes research, community‐based participatory research) that have community, patient, and/or stakeholder engagement, feedback, and dialogue as core principles’ [1]. One of the key elements of stakeholder engagement in research is the assessment of important issues that the population of interest (stakeholders) care about, and this information can then inform the decision‐making about research topics and directions [2]. From an academic perspective, engaging stakeholders to identify important issues to inform research decisions may be particularly important for projects with a wide scope for possible research, a large availability of potential data to analyse, and multiple research questions that require prioritisation. It is also important to ensure research addresses an issue that is a priority for relevant stakeholders, making it of real‐world importance rather than just an academic exercise. Therefore, by engaging stakeholders in informing research directions, it may help to both increase relevance and interest of research, reduce barriers to implementation and enhance data quality and value [2, 3].

It is important that stakeholder engagement is co‐produced with Patient and Public Involvement and Engagement (PPIE), or through public contributors—often used interchangeably with PPIE—referring to members of the public, including patients, carers and people with lived experience, who are involved in research through PPIE activities. The NIHR defines co‐production as ‘an approach where researchers, practitioners and members of the public work together, sharing power and responsibility from the start to the end of the project, including the generation of knowledge’ [4]. Involving members of the public in co‐produced research is important to improve quality, outcomes and applicability of research [5], and involving them within the co‐production of stakeholder engagement can help to ensure that research remains grounded in reality and maintains a person‐centred perspective [6]. Co‐production involves public contributors being embedded in all stages of the research process, and working collaboratively to identifying research questions, design and priority setting, governance, co‐delivery of research activities, communication of key findings and involvement in knowledge exchange such as sharing information, insights and expertise between different groups [7].

The MELD‐B project [8] aims to identify early life targets until age 18, for the prevention of early‐onset, burdensome multiple long‐term conditions (MLTCs) and to characterise ‘burden’ for people living with MLTCs. Given the potential scope of exposures across the childhood life‐course, and the potential conflicting time points for prioritisation, we considered the integration of PPIE and wider stakeholder engagement as crucial for the planning of the MELD‐B project. We identified that drawing upon the experiences, expertise and knowledge of patients, public and wider stakeholders would help to enrich discussions, steer our academic analysis, inform research decisions, and contribute to meaningful and relevant outcomes. Identifying and addressing priorities in childhood for future MLTCs can also help to develop effective prevention strategies.

In pursuit of this collaborative approach, in previous research we sought the views of public contributors including young adults (age 18–30) regardless of MLTC status and older adults (age 40–65) living with self‐reported MLTCs, to design a conceptual framework identifying domains of risk in early life for future MLTCs [9]. This study was achieved through two co‐produced focus groups, and 20 public contributors from a range of demographic backgrounds (age, gender, ethnicity and geographical location) attended the focus group sessions [9]. Combining the findings from these focus groups with a scoping policy and research evidence review, 12 domains of early life risk for future MLTC were identified [9]. These domains incorporated personal, social, economic, behavioural and environmental factors, adverse childhood experiences (ACEs), socio‐economics, the social and physical environment, and education. This study found that policy recommendations more often focused on individual‐level factors as opposed to the wider determinants of health discussed within the research evidence. Some domains discussed with public contributors, such as religion and spirituality, health screening and check‐ups, and diet, were not adequately considered within the research evidence or policy [9].

Building on the conceptual framework findings, we wanted to take a further collaborative approach with the aim of identifying critical time points and targets in the early life‐course for feasible and practical interventions to prevent or delay MLTCs. Therefore, a stakeholder workshop, co‐designed with PPIE colleagues, was conducted to steer the MELD‐B research study [8]. Such co‐produced initiatives are imperative considering the escalating prevalence of MLTCs [10] that often occurs earlier in the life‐course among people from more socio‐economically disadvantaged backgrounds [8]. We know that factors across the adult life‐course such as demographic characteristics (e.g., age and ethnicity) [11, 12], behaviours (e.g., smoking, diet, alcohol consumption and physical activity) [13] and broader environmental and social experiences (e.g., their education, work and income) [14, 15] influence the chances of developing MLTCs. Yet, the influence of childhood exposures on MLTCs has not been fully explored. Moreover, the timing and nature of childhood exposures to various factors and determinants may significantly influence earlier and more extensive accumulation of long‐term conditions.

To meet the significant challenge of preventing or delaying MLTCs, there is a need to take a life‐course approach to understand the influence of wider determinants in childhood on the sequence of MLTC accrual, and the expertise and experiences of stakeholders are crucial for informing this study. Therefore, the aims of the MELD‐B stakeholder workshop were to:

1. Guide the MELD‐B research study on research priorities for early life prevention of MLTCs.
2. Identify targets and time points in childhood for interventions to prevent or delay the development of MLTCs.

This paper discusses how the stakeholder workshop was designed and conducted, the findings from the workshop, and how the findings are informing the next steps of the MELD‐B project.

## 2 Methods

### 2.1 Co‐Designing the Workshop With PPIE

Public contributors played a key role in shaping the workshop. They helped identify stakeholders to invite and co‐produced the agenda, including its structure and format. They also contributed to the planning and design of the PowerPoint slides used to guide the workshop. As part of these slides, we included a co‐designed, project‐specific animation [16] developed earlier in the MELD‐B project. Public contributors were involved in reviewing the storyboard, advising on how to translate it into animation, and providing voiceovers.

Additionally, public contributors co‐designed two fictional personas (Supporting Materials Figure 1) used to frame the research. They contributed ideas about childhood experiences that might influence health and helped map these into timelines across childhood.

All public contributors were invited to attend the workshop and participate in both full‐group and breakout discussions, sharing their lived experiences. Following the workshop, they reviewed the themes identified by the research team and contributed to refining them. Public contributors were also involved in disseminating the findings, including this paper. Two are named co‐authors, while two others chose not to be named.

### 2.2 Recruitment of Stakeholders

The research team and PPIE colleagues identified and invited stakeholders, and then additional stakeholders were identified through snowball sampling. The inclusion criteria included any stakeholder who had professional expertise and experience working with children or families. Effort was made by the research team to ensure that the stakeholders represented the whole of the child life‐course, for example, people involved in perinatal care, early years, teenagers and young adults.

### 2.3 Conducting the Workshop

The workshop was held online via Microsoft Teams [17] and lasted 2 h. Stakeholders were divided into three breakout groups, each intentionally mixed to ensure diverse perspectives. Each group was supported by two facilitators. Two public contributors attended the workshop and helped facilitate the full‐group discussions. Due to limited numbers of public contributors, one breakout group did not have a public contributor present.

Jamboard [18] (an online interactive whiteboard) was used to collate ideas, and both the stakeholders and facilitators could access and add to the Jamboard throughout the workshop. Following the breakout rooms, stakeholders were convened into a single group for a full‐group discussion. One facilitator within each breakout group and at least one facilitator in the full‐group discussion collated notes, and these were added to the Jamboard.

A poll was administered via Vevox (a live poll directly embedded into PowerPoint presentations) at the end of the workshop giving choices to prioritise time points for interventions. The poll included four time points (birth, 5/7, 10/11 and 16 years), and these time points were selected as they aligned with the time points being considered for analysis within the MELD‐B project.

### 2.4 Identifying Themes and Dissemination

Notes from the breakout and full‐group discussions were collated, and initial themes were generated by one team member (S.S.). These themes, along with the full notes and recordings, were shared with the wider team, including public contributors. All were invited to review and comment on the themes, and through follow‐up meetings, the themes were refined collaboratively.

With respect to analysis and writing up, initial drafting of the academic paper was undertaken by two team members (S.S. and N.A.). All other team members, including public contributors, were invited to contribute to the writing process by reviewing and editing drafts. All feedback was considered equally.

### 2.5 Reflexivity

N.A. has a clinical background and is a subject expert in public health, life‐course epidemiology and health inequalities. S.S. is a postdoctoral research fellow with expertise in life‐course epidemiology with expertise in childhood. S.F. is an academic MLTCs subject expert and has experience of caring for people with MLTCs in general practice. J.M. and J.W. are PPI members with personal experience of living with LTCs. Other PPI members have experience of living with MLTCs. R.W. is a public health consultant in a local authority whose portfolio includes physical activity and food. J.G. is a specialist public policy officer with expertise in public health and maternal and early childhood development policies. The authors' professional and lived experiences meant the process of designing the workshop was informed by multiple perspectives. N.A. and S.S. presented the goal of the proposed workshop in terms of informing the data analyses. Discussions with our MLTC lived experience public contributors centred around how best to achieve this goal from the proposed stakeholder workshop and what questions to ask. Our public contributors also suggested the use of a project‐specific animation and imaginary personas to help frame our research questions. The research team members and the public contributors were asked to suggest stakeholders to approach for the workshop and invited to attend and contribute to the workshop.

Further, two of the public contributors have provided their own personal reflections on working on the MELD‐B project below.

Public contributor 1: ‘*Since becoming part of MELD‐B's PPI contributors in 2022, I have actively supported 2 of the work packages (4 and 5) to offer both personal experience and tangible input into the study's development. In respect of WP4, I have directly influenced the conversations about how we should be thinking about milestones that affect childhood development and interventions that can affect the course of their life. I feel that our conversations with researchers have been respected and that we can share ideas as equals. I am also delighted to be the voice of the official animation for the study, as well as being a co‐author for policy papers on how decision makers can make a difference to the lives of individuals with multiple long‐term conditions’.*

Public contributor 2: ‘*I have supported MELD‐B for over 5 years, as a PPIE co‐author and project advisory group member participant. I remain an advocate of the project, given I appreciate the benefit data provides in identifying the causal paths to a worsening condition that arises over time, if not prevented at the earliest possible occurrence. Engaging with the team throughout has been collaborative, given I have helped shape the deliverables within many of the work streams, advising upon the clarity and tone of voice within reports published by the team: Confirming the vision we had at the outset of the project 5 years ago’*.

## 3 Findings

25 stakeholders from backgrounds including integrated care boards (*n* = 5), healthcare practitioners (*n* = 3), academics (*n* = 4), council employees (*n* = 9) and not‐for‐profit organisations (*n* = 4) attended the workshop and contributed in an advisory capacity.

**Figure 1:**
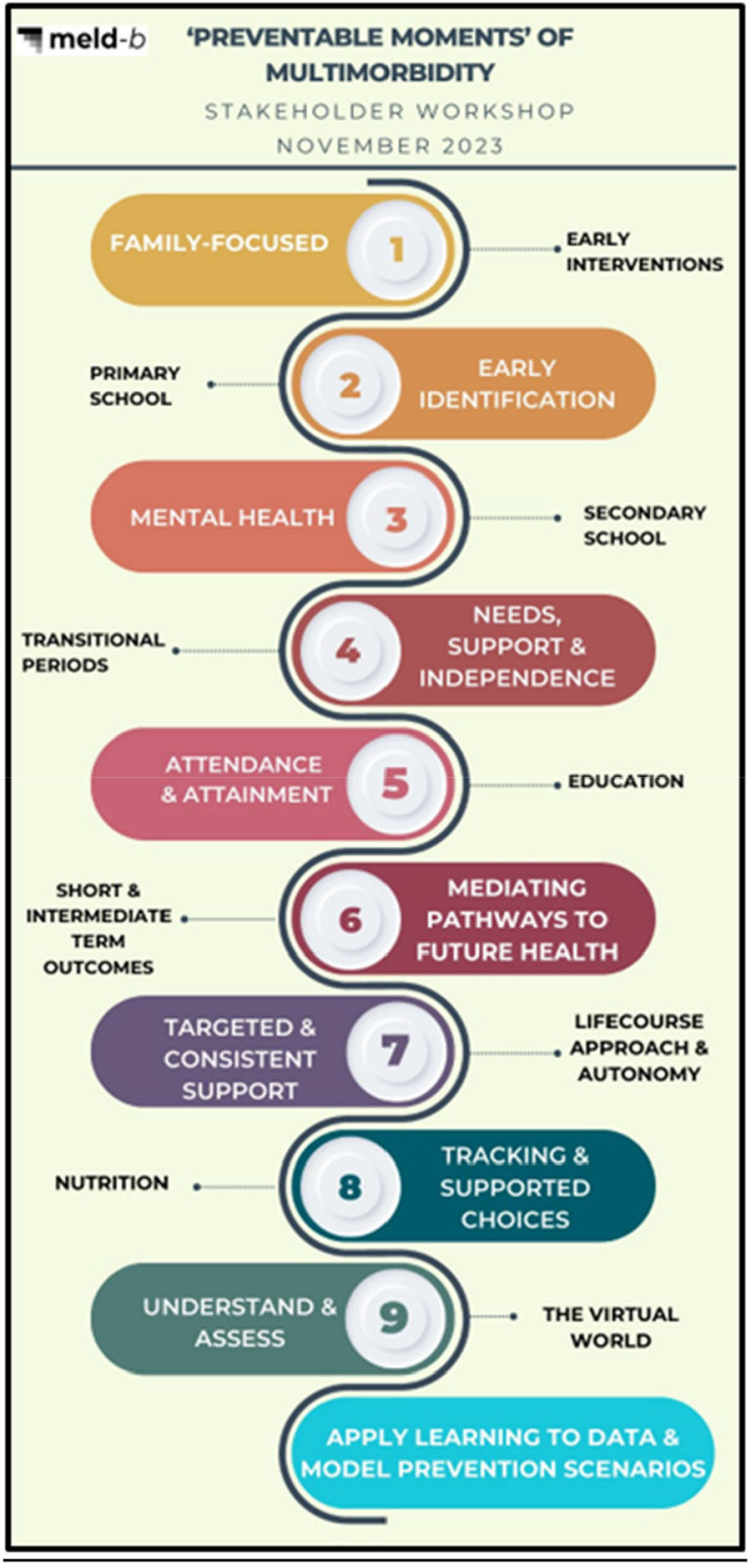
Findings from a stakeholder workshop to inform MLTC prevention research.

The findings from the workshop can be summarised under nine themes and sub‐themes; these are highlighted in Figure 1, and a short summary of the themes are discussed below.

### 3.1 Family Focused

#### 3.1.1 Early Interventions

Stakeholders discussed how parents often have the sense ‘it won't happen to them’, resulting in them not always understanding the links between current choices and the long‐term health impacts of the choices they are making. A perception of the stakeholders was that for many families the focus is on ensuring there is enough food available rather than the lifelong impacts of current dietary decisions. Interventions are about changing family mindsets; it is not just about teaching children about health in school, but key messages should be communicated to parents, through transgenerational transmissions of thoughts and behaviours. However, this might not always be parents influencing their children; children can influence the choices of their parents.

Identification and intervention of issues during the first few years of life allows for tailored support across the rest of childhood. Early inventions may provide the opportunity to change habits and trajectories at an earlier point, especially given that as children grow older these habits and trajectories might become harder to change. During the early years, the focus should be on the family in particular the parents or the primary caregiver given it is these individuals who will have the greatest influence on a child.

### 3.2 Early Identification

#### 3.2.1 Primary School

The stakeholders discussed how earlier diagnosis and identification could mean a child and their family can become more aware of their own condition(s), in turn developing a better understanding of how to support themselves. Early diagnosis and identification of both single long‐term conditions in childhood that might impact health across the life‐course, and also neurodiverse conditions should mean support and assistance can be tailored towards a child at an earlier stage in the life‐course. However, post diagnosis support is often limited, and children regularly wait a long time to receive the correct support and assistance.

Primary school represents a good opportunity for intervention given this is when issues first become apparent and is the first universal touchpoint for children. Interventions in primary school are likely to require a whole family approach and therefore issues with engagement can often be complex.

### 3.3 Mental Health

#### 3.3.1 Secondary School

The stakeholders discussed how many issues, particularly related to mental health, would have already ‘*manifested’* themselves by secondary school age, and therefore it becomes a matter of *‘firefighting’* problems. At this age, there is a shift in emphasis from factors involving the family environment (important during primary school years), towards the impact of wider environmental factors. Therefore, interventions during secondary school need to be less targeted at the whole family but rather aimed at the individual child, given children have more autonomy over their own choices and decisions. It is also important to consider aspects outside of school, and this is especially salient for secondary school age children who have more independence over these out‐of‐school choices.

### 3.4 Needs, Support and Independence

#### 3.4.1 Transitional Periods

Transitional periods were defined by the stakeholders as times when a child may experience high levels of change. These periods include going into primary school and transitioning from primary to secondary school, given that support around these period changes considerably. In relation to mental health, earlier identification and interventions during these transitional periods, that is, at the start of secondary school, could help reduce the build‐up of issues that often become apparent when child reaches a *‘crisis’* point later in secondary school. Additionally, identification of undiagnosed special educational needs at transitional ages would allow for the support and interventions to be put in place for the remainder of childhood. A later transitional period includes the transition to employment. This period represents an opportunity where new habits can be learned and adopted, and these habits might have a large influence on life trajectories.

### 3.5 Attendance and Attainment

#### 3.5.1 Education

There was a strong focus on education during the discussions. Schools represent a universal touchpoint for potential interventions. However, the school curriculum is not focused on supporting health and well‐being, suggesting a shift in model is needed.

*‘For as long as schools are judged on English and Maths results then everything else (including health and wellbeing) will fall behind that.’*

There is also a strong correlation between worse attendance and poor attainment, which in turn will likely influence health. Education attainment is often used as a proxy indicator for a wider range of factors throughout childhood. However, although educational settings offer a good opportunity for interventions, if these interventions are unable to be operationalised outside of school, that is, within the wider home environment, then the intervention is likely to have little impact.

### 3.6 Mediating Pathways to Future Health

#### 3.6.1 Short‐ and Intermediate‐Term Outcomes

The stakeholders expressed how it is important to consider shorter‐term or intermediate outcomes as well as longer‐term health outcomes. They noted that this may be particularly important for influencing policy, given it is easier to engage policymakers on short or intermediate outcomes opposed to outcomes much later on in the life‐course. Therefore, interventions in childhood should not just focus on preventing health at a later point in the life‐course, but interventions should also consider improving shorter‐term outcomes that may be on the mediating pathway to health later in life. Examples discussed included school attainment, school attendance, education, jobs and employment.

### 3.7 Targeted and Consistent Support

#### 3.7.1 Life‐Course Approach and Autonomy

During the discussions, the concept of a life‐course approach to childhood manifested in two contrasting approaches: (1) a targeted approach, that is, making sure a child has the right interventions at the right time, and (2) supporting a child throughout childhood, that is, not just when children present with symptoms or issues. Despite stakeholders identifying the need to take a life‐course approach, it was also discussed how this approach is often not reflected by families. A perception of the stakeholders was that parents often do not consider the lifelong impacts of choices made in childhood but rather the focus is on short‐term impacts and the next important child milestone (i.e., going to preschool, school, etc.). At a more basic level, it was discussed how for some families the focus is on ensuring there is enough food for a child's next meal and therefore thinking about lifelong impacts of current decisions would not be a consideration.

### 3.8 Tracking and Supported Choices

#### 3.8.1 Nutrition

Diet and obesity were discussed widely across the three stakeholder breakout groups, and it was noted that obesity and tooth decay are a big issue in Southampton currently. A perception of the stakeholders was that many decisions around food stem from socio‐economic factors, and the focus is often on how much a family can afford and what will sustain a child in the short term, rather than what is healthy. There is a lack of support around introducing children to solid foods, and it is important to track obesity beyond the early years (age 5–11), especially given the rise in overweight and obesity during this period. As children get older, they have more autonomy over the choices they make around food; however, earlier in the life‐course, the focus on healthy eating falls to the parents.

### 3.9 Understand and Assess

#### 3.9.1 The Virtual World

The stakeholders discussed how the virtual environment is important both for physical and mental health. For many children, the virtual environment may now play a bigger role on influencing choices, habits and behaviours compared to the physical environment. One of the challenges with the virtual environment is that it is often outside the scope of parent's knowledge and understanding, and so it is difficult to regulate.

#### 3.9.2 Findings From the Poll

**Figure 2:**
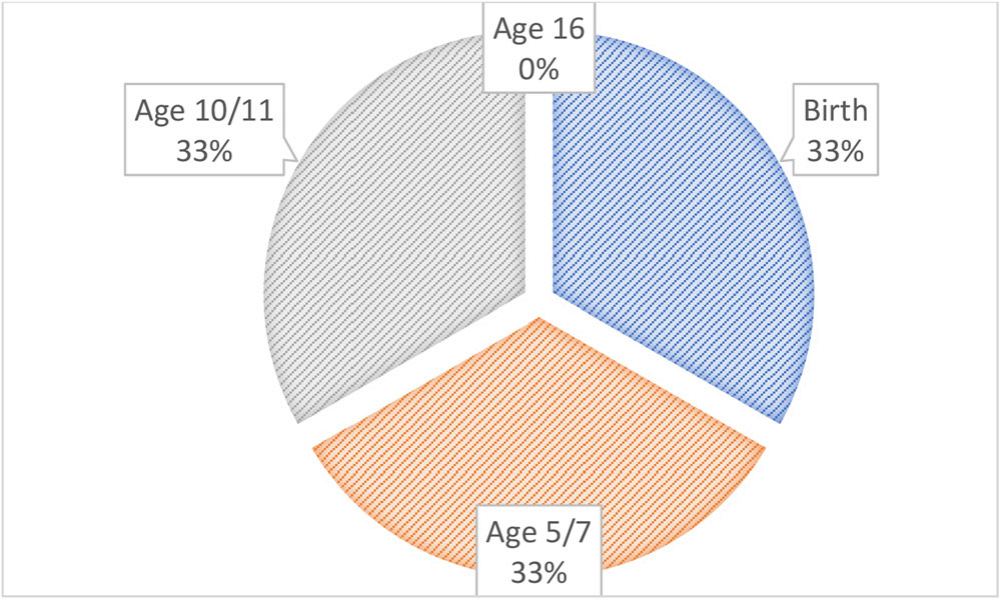
A pie chart showing which time points should be prioritised for interventions (*n* = 18).

At the end of the workshop stakeholders were asked ‘if they had to prioritise one time point in childhood (birth, age 5/7, age 10/11 and age 16) what timepoint would it be?’. Figure 2 summarises the results. As shown, there was an equal split of votes for three of the time points. Important time points for interventions included transitional ages such as the start of primary (age 5/7) and secondary school (age 10/11) and the very early life‐course (birth). Late adolescence (age 16) was considered a less important time point than these earlier time points.

## 4 Discussion

Through the workshop, we heard from stakeholders from various backgrounds including NHS integrated care board, healthcare practitioners, academics and not‐for‐profit organisations about the key periods in early life where there is opportunity to intervene to prevent or delay MLTCs. Themes discussed included: mental health, educational attainment and attendance, the early identification of health conditions and neurodiversity, tracking and supporting nutritional choices, the importance of transitional periods, understanding emerging factors such as the virtual world, and the importance of considering intermediate outcomes that may be on the pathway to future ill health. Stakeholders suggested that family‐targeted interventions maybe more important to prioritise in early childhood; however, at secondary school age, individual‐focused interventions may become more significant. The poll that included four time points (birth, 5/7, 10/11 and 16 years) identified—by an equal split—the first three as the most important time points for interventions, with ages 5/7 and 10/11 years considered transitional ages at the start of primary and secondary school, respectively. Later adolescence (age 16 years) was not voted as a priority compared to the other time points.

The findings from the workshop have begun to be implemented across the MELD‐B project. Firstly, the findings from the workshop validated our earlier conceptual framework that characterised the population‐level domains of early‐life determinants of future MLTC risk [9]. For example, the stakeholders discussed the family environment, education, child health (including mental health), diet and nutrition, and the intergenerational transmission of thoughts and behaviours—these factors are all included within the conceptual framework [9]. However, we acknowledge that based on the stakeholder workshop, a 13th domain should be considered. This domain would focus on the virtual world as an emergent determinant for future health outcomes. Therefore, when planning future research, we intend to amend the domains we consider to include the role of the virtual world.

The workshop has informed the time points to focus on within the MELD‐B project, and as such, we have been concentrating on the transitional time periods identified from the workshop. For example, we have focused a number of academic papers on conceptualising early life exposures (linked to the conceptual framework [9], and the themes identified from the stakeholders) recorded at birth and age 10/11 on the risk of developing MLTCs including obesity and hypertension comorbidity [19, 20], a count of MLTCs, and MLTC with role limitations. We are additionally exploring the relationship between predictors (linked to the conceptual framework [9] and the themes identified from the stakeholders), this time recorded at age 5, and MLTCs with role limitations in midlife. Additionally, as a result of the stakeholder workshop, the MELD‐B project took the decision not to focus on later adolescence (age 16 years) as it was not voted as a priority by the stakeholders (compared to the earlier time points).

As part of the MELD‐B project, we are focusing some of our research on the specific themes identified by the stakeholders. One of the themes discussed by the stakeholders was the importance of attendance and attainment within the wider context of education. Therefore, using a cohort of children born in Aberdeen, Scotland, we have been exploring the relationship between education and academic ability in childhood, focusing on variables including school attendance, school test scores and educational difficulties (such as dyslexia), on the number of outpatient appointments and hospital appointments attended in adulthood [21].

The MELD‐B project was intended to consider childhood determinants of further health outcomes in the adult life‐course. However, the stakeholder workshop has informed us that it is important to consider short‐/intermediate‐term outcomes as well as longer‐term health outcomes. Interventions in childhood should not just focus on preventing health at a later point in the life‐course, but interventions should also consider improving shorter‐term outcomes that may be on the pathway to health. As a result, we have begun to consider some of these short‐/intermediate‐term outcomes; for example, we are exploring childhood predictors of childhood obesity, as childhood obesity is on the pathway to future health outcomes including diabetes, depression and reduced health‐rated quality of life [22].

Stakeholders identified transitional periods in early childhood (age 10/11 and younger) as critical time points for prevention interventions, aligning with a well‐established body of literature that defines and explores the significance of these early‐life developmental stages [23, 24, 25, 26]. However, some empirical findings diverge from stakeholder perspectives. For instance, stakeholders emphasised early childhood (before age 10/11) as more influential than later adolescence. In contrast, Graf et al. [24] found that ACEs occurring during adolescence (ages 13–17) were more strongly associated with disrupted transitions to adulthood including poorer outcomes in education, employment, criminal justice involvement and psychiatric diagnoses than ACEs occurring in earlier periods (ages 0–2, 3–5 and 6–12).

Nevertheless, other research supports the stakeholders' emphasis on earlier transitions. For example, the transition from primary to secondary school has been identified as a particularly vulnerable period for outcomes related to education, well‐being and employment [25]. Similarly, the transition into formal schooling from preschool or nursery settings often framed in terms of ‘school readiness’ has been linked to later academic, emotional and behavioural outcomes [26].

These findings suggest that the significance of early‐life transitional periods may be context‐specific, depending on the type of early life exposure and the outcomes under consideration. Further, these transitional periods align with the Life Course Health Development (LCHD) framework [27, 28], which emphasises the importance of ‘critical stages’ across early life for future outcomes across the life‐course, with a particular focus on the prenatal period; birth, neonatal period and infancy; childhood; and adolescence, in shaping long‐term health and well‐being.

Finally, we are working with public contributors to co‐design the most effective methods for communicating with stakeholders how their input is being integrated into, and influencing, our research. Working with the same group of contributors, we are now organising a follow‐up workshop. This workshop will provide an opportunity to share the themes identified in the initial session, gather feedback, explain how participants' input is shaping the ongoing MELD‐B project, and identify areas for future research. Additionally, whilst the focus of this study was to gain the perspectives of stakeholders working with young people and children, we acknowledge that in future workshops the perspective of children and young people should additionally be explored.

## 5 Strength and Limitations

One of the strengths of this workshop was our ability to engage a diverse group of stakeholders with expertise across various time points in childhood. Additionally, we successfully integrated public contributors at every stage of the research, from initial development to dissemination. However, the workshop has limitations, including a small number of stakeholders and the fact that we only conducted one workshop. Moreover, due to the connections within the research team, the stakeholders were primarily from the South East region of England. As a result, stakeholders from other regions of England and the United Kingdom might have different priorities based on their regional issues and needs.

## 6 Conclusion

Successful implementation of a co‐designed stakeholder workshop has led to the identification of themes that can be used to shape research questions and prioritise data analysis around potential factors and time periods that could have an impact on the development of MLTCs. Ultimately, this workshop is guiding the early life workstream of the MELD‐B project in terms of analyses and outputs, and we hope that the results can help support the development of effective policies and practices for preventing and reducing MLTCs.

## Author Contributions

**Sebastian Stannard**, **Rebecca Wilkinson**, **Jaskiran K. Gill**, **James, McMahon**, **Jack Welch**, **Simon D. S. Fraser**, and **Nisreen A. Alwan:** conceptualization. **Sebastian Stannard**, **Nisreen A. Alwan**, **Jaskiran K. Gill** and **Rebecca Wilkinson:** data curation. **Sebastian Stannard:** formal analysis. **Nisreen A. Alwan:** visualization. **Sebastian Stannard** and **Nisreen A. Alwan:** writing – original draft. **Sebastian Stannard**, **Rebecca Wilkinson**, **Jaskiran K. Gill**, **James**, **McMahon**, **Jack Welch**, **Simon D. S. Fraser**, and **Nisreen A. Alwan:** writing – reviewing and editing. All authors approved the final manuscript.

## Disclosure

The views expressed are those of the authors and not necessarily those of the NIHR or the Department of Health and Social Care.

## Ethics Statement

The authors have nothing to report.

## Consent for Publication

The authors have nothing to report.

## Conflicts of Interest

The authors declare that they have no competing interest.

## Supporting information


**upplementary Figure 1:** Imaginary persona to frame the research question.

## Data Availability

All data collected is reported within this paper.
