# Supplementary material for: A Co‐Produced Stakeholder Workshop to Identify Key Time Points and Targets for Life‐Course Prevention of Multiple Long‐Term Conditions
Source: Health Expect. 2025 Oct 24;28(5):e70475. doi: 10.1111/hex.70475 (PMC12550862; doi:10.1111/hex.70475)
Supplement: Supplementary file 1 — upplementary Figure 1: Imaginary persona to frame the research question. [file HEX-28-e70475-s001.docx]

**Supplementary Materials**

*
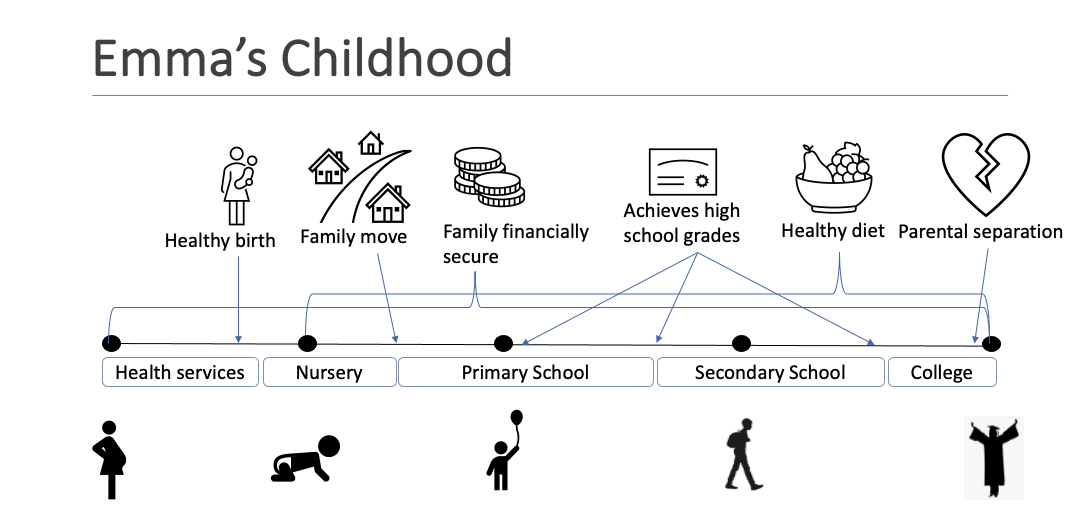
Supplementary Figure 1: Imaginary persona to frame the research question*

*
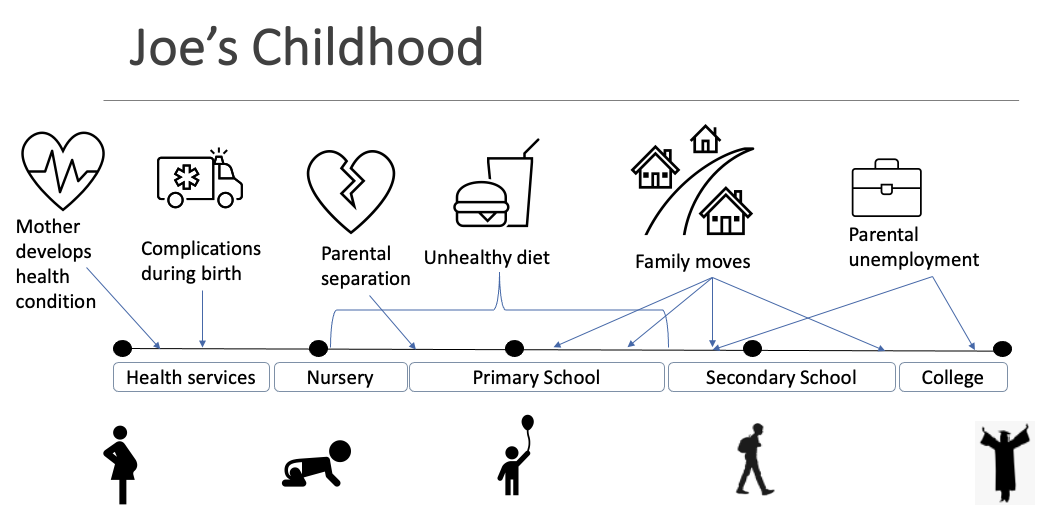
*
